# Experimental and Computational Analysis of a Large Protein Network That Controls Fat Storage Reveals the Design Principles of a Signaling Network

**DOI:** 10.1371/journal.pcbi.1004264

**Published:** 2015-05-28

**Authors:** Bader Al-Anzi, Patrick Arpp, Sherif Gerges, Christopher Ormerod, Noah Olsman, Kai Zinn

**Affiliations:** 1 Division of Biology and Biological Engineering, California Institute of Technology, Pasadena, California, United States of America; 2 Department of Molecular Biology, Princeton University, Princeton, New Jersey, United States of America; 3 Division of Physics, Mathematics, and Astronomy, California Institute of Technology, Pasadena, California, United States of America; 4 Control and Dynamical Systems Option, Division of Engineering and Applied Sciences, California Institute of Technology, Pasadena, California, United States of America; National University of Singapore, SINGAPORE

## Abstract

An approach combining genetic, proteomic, computational, and physiological analysis was used to define a protein network that regulates fat storage in budding yeast (*Saccharomyces cerevisiae*). A computational analysis of this network shows that it is not scale-free, and is best approximated by the Watts-Strogatz model, which generates “small-world” networks with high clustering and short path lengths. The network is also modular, containing energy level sensing proteins that connect to four output processes: autophagy, fatty acid synthesis, mRNA processing, and MAP kinase signaling. The importance of each protein to network function is dependent on its Katz centrality score, which is related both to the protein’s position within a module and to the module’s relationship to the network as a whole. The network is also divisible into subnetworks that span modular boundaries and regulate different aspects of fat metabolism. We used a combination of genetics and pharmacology to simultaneously block output from multiple network nodes. The phenotypic results of this blockage define patterns of communication among distant network nodes, and these patterns are consistent with the Watts-Strogatz model.

## Author Summary

We discovered a large protein network that regulates fat storage in budding yeast. This network contains 94 proteins, almost all of which bind to other proteins in the network. To understand the functions of large protein collections such as these, it will be necessary to move away from one-by-one analysis of individual proteins and create computational models of entire networks. This will allow classification of networks into categories and permit researchers to identify key network proteins on theoretical grounds. We show here that the fat regulation network fits a Watts-Strogatz small-world model. This model was devised to explain the clustering phenomena often observed in real networks, but has not been previously applied to signaling networks within cells. The short path length and high clustering coefficients characteristic of the Watts-Strogatz topology allow for rapid communication between distant nodes and for division of the network into modules that perform different functions. The fat regulation network has modules, and it is divisible into subnetworks that span modular boundaries and regulate different aspects of fat metabolism. We experimentally examined communication between nodes within the network using a combination of genetics and pharmacology, and showed that the communication patterns are consistent with the Watts-Strogatz topology.

## Introduction

Systems biology explores the emergence of patterns, structures, and properties in biological systems that cannot be understood by examining individual components. One of the goals of this discipline is to discover how the topological arrangements of proteins within signaling networks endow the networks with features that are important for their cellular functions.

Due to the availability of extensive proteomic data and the existence of strain collections bearing deletion mutations for most of its genes, the budding yeast *Saccharomyces cerevisiae* is an excellent system in which to discover topological principles governing the design of signaling networks [1,2]. Some network analyses in yeast have examined all of the proteins identified by genome-wide proteomic methods [3–15], while others have focused on essential genes that encode highly connected proteins, referred to as hubs, that are characterized by a lethal phenotype when removed [16–18]. There are disadvantages associated with using either of these approaches to analyze the relationships between network topology and function. First, although proteomic data define connections among proteins, not all connections made by a given protein are relevant when that protein performs its functions in a specific cellular process. Second, lethality can be produced through many different mechanisms, so genes and proteins required for viability do not necessarily have related functions. Third, the contributions of essential genes to survival can only be scored as viability or lethality. Most biological processes, however, exhibit variations in output strength, and incorporation of this information can add value to network models. Fourth, due to the lethal phenotype of these genes, networks of essential genes usually do not provide information about their relationships to the products of interacting nonessential genes.

Here we show that molecular mechanisms used for regulation of fat storage in yeast provide an excellent system for network analysis. First, the mutant phenotype, an alteration in fat levels, is specific enough to suggest that there should be molecular relationships among many of the proteins in the network. Second, the severity of the fat storage defect when a fat level-regulating protein is removed can be quantitatively assessed, and this can be used to determine the protein’s importance to network function. Third, since the loss of a fat storage-regulating gene usually does not cause lethality, mutants selected for quantitative changes in fat content can also be assayed for alterations in other aspects of fat metabolism, such as lipid droplet (LD) morphology and the ability to use different carbon sources for fat synthesis. By using a system-wide approach that combines genetic, proteomic, pharmacological, mathematical, and physiological analysis, we have identified and characterized a physically interconnected network of 94 proteins that regulates fat storage in budding yeast.

The fat regulation network is not scale-free, and is best approximated by the Watts-Strogatz model [19], which generates “small-world” networks with high clustering and short path-lengths. Such networks have many features that are useful for biological control. The importance of a protein to network function is dependent on a particular kind of topological centrality, and the use of this centrality measure may provide a guideline for future analysis of proteins in other biological networks. We were also able to validate the network model by experimentally blocking function of multiple network nodes and showing that the patterns of internode communication predicted by this analysis are consistent with the small-world architecture of the network.

## Results

### Identification of a large set of yeast genes for which mutations increase fat content

**Fig 1:**
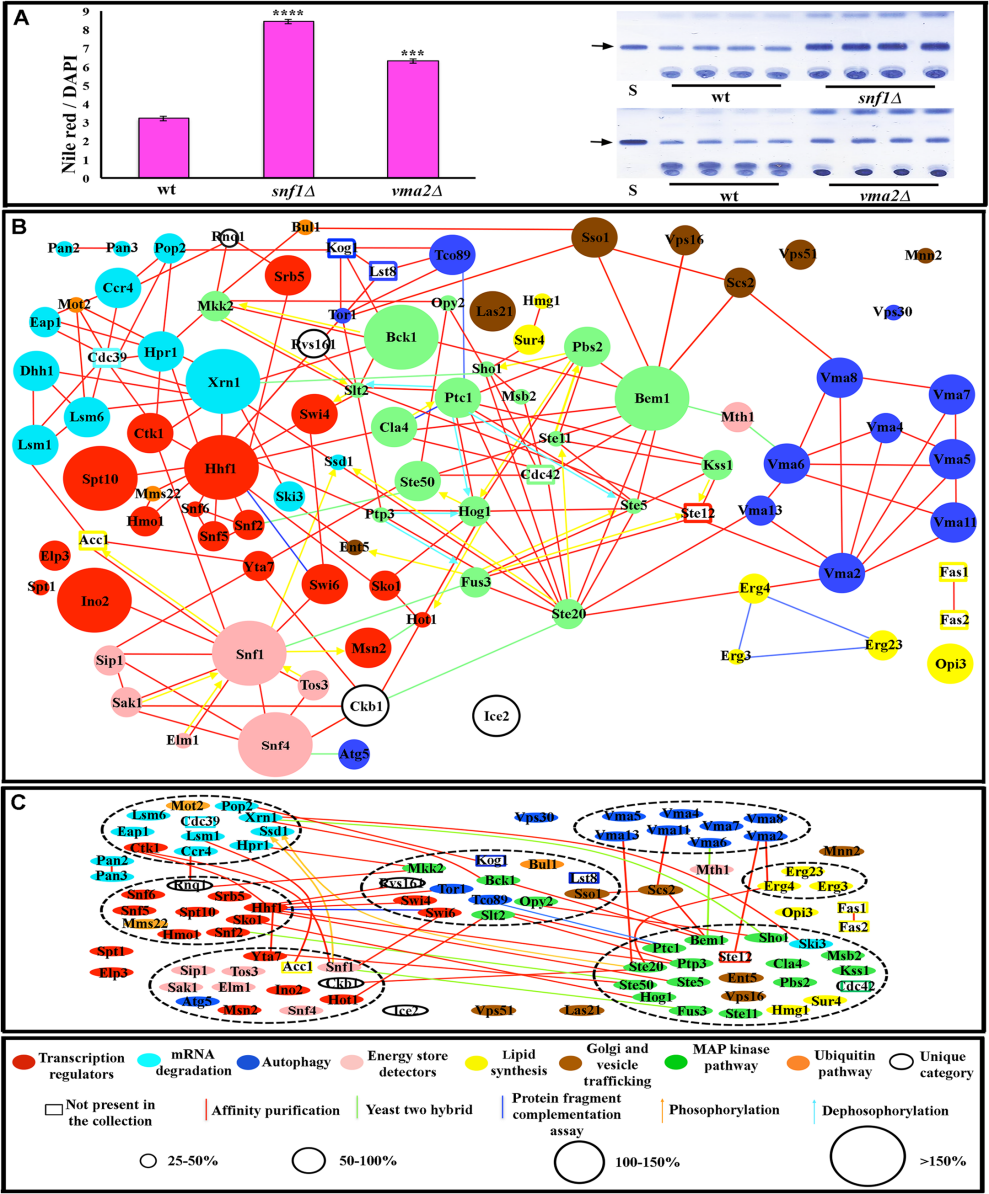
The fat storage regulation network. (A) Nile Red/DAPI measurements (left), and TLC analysis (right) for two mutants (four replicates for each). S is lard standard, and the arrow indicates the triglyceride band. Four replicate samples are shown. (B) Fat storage-regulating yeast genes encode connected proteins. In the key at the bottom, the sizes of the circles represent the magnitude of the increase in fat levels relative to wild type, as measured by TLC (for quantification of the fat levels in each mutant see S1 Table). The molecular function of a given protein is represented by the color of the circles, while the type of reported molecular contact between two proteins is represented by the color of the connecting lines. Unfilled rectangles are proteins encoded by 8 essential genes that are likely to be part of the network (see text). (C) The network contains 7 highly connected protein communities composed of more than three proteins (enclosed by black dashed lines). Error bars in (A) are standard deviations of 10 replicas for a given genotype, and asterisks denote t-test statistical significance as compared to wild-type (wt) (**p < 0.5E-5, ***p < 0.5E-8, ****p < 0.5E-9).

We developed a quantitative 96-well plate assay to screen the viable *Saccharomyces cerevisiae* deletion collection for alterations in fat content. In this assay, stored fat levels in fixed yeast cells were assessed by staining with the lipid dye Nile Red together with the nuclear dye DAPI and measuring the Nile Red/DAPI fluorescence ratio. Positive mutants were confirmed using a thin layer chromatography (TLC) assay to measure triglycerides, as described by [20](Fig 1A) and by histological staining of fixed cells with another fat-specific dye, BODIPY 493/503. Mutations in 86 genes caused statistically significant increases in fat content (Fig 1 and S1 Table).

54 of the 86 genes identified in this screen have metazoan orthologs or relatives. Of these, *brahma* (a chromatin remodeling protein orthologous to *SNF2*), *histone H4* (*HHF1* ortholog), *cdk12* (a Cdk family kinase orthologous to *CTK1*), and *me31b* (an RNA helicase orthologous to *DHH1*), were identified in one or both of the published screens of cultured *Drosophila* cells for LD morphology phenotypes[21,22].

### The proteins encoded by fat-regulatory genes define a highly interconnected network

Extensive proteomic data exist for budding yeast (see [10–13,23]). These data were obtained by a variety of methods, including the two-hybrid system[3,9], the protein fragment complementation assay[4], affinity purification and co-precipitation[7,8], and analysis of global protein phosphorylation patterns[5,6]. We assembled current data on physical interactions for proteins encoded by these genes from the *Saccharomyces* Genome Database (www.yeastgenome.org) and BioGRID Database (biogrid.org). Remarkably, superimposition of our genetic data on these proteomic data showed that 91% of the mutations we identified affect genes encoding proteins that have physical connections to one another, forming a densely interconnected network (Fig 1B). Most of the proteomic interactions shown in the figure were defined by affinity purification studies, indicating that they define stable and abundant complexes that exist *in vivo*.

The network defined by screening the viable deletion collection necessarily contains only nonessential genes. However, proteins encoded by essential genes are also known to be involved in regulation of fat synthesis and storage, or are components of pathways identified by the screen of nonessential genes (see next section for discussion of network pathways). We thus added eight proteins encoded by essential genes to the network. Fas1 and Fas2 are subunits of fatty acid synthetase, and Acc1 is acetyl-coA carboxylase. These enzymes are required for *de novo* synthesis of long-chain fatty acids. Kog1 and Lst8 are components of the TOR complex 1 (TORC1), which we identified as part of the network through the identification of two other TORC1 subunits, Tor1 and Tco89. Ste12 is a transcription factor activated by a MAPK signaling cascade, and Cdc42 is a small G protein that regulates MAPK signaling. Many of the genes in the network encode MAPK pathway components. Cdc39 is a component of the CCR4-NOT core complex, and we identified many other components of this complex in our screen [24–28]. These essential proteins were added in order to create a more complete and biologically meaningful network, not to artificially increase its connectedness by adding “hubs”. In fact, only two of these eight proteins, Cdc39 and Cdc42, have more than four connections to other proteins in the network. Moreover, as described below, we have shown that removal of all of these essential proteins from the network does not significantly affect its topological properties. There are 94 proteins (nodes) and 203 total connections (edges) in the complete network (S2 Table).

To evaluate the significance of this network, we first wished to determine whether the extensive interconnections among its proteins reflect a common biological function and are likely to have been selected by evolution. To address this issue, we asked whether randomly selected collections of similar numbers of proteins from the yeast proteome would display similar connection densities. We generated 200 random collections of approximately 98 proteins (nodes) each and annotated the interactions within each collection that had been identified in published experiments. Of the 200 networks thus defined, most had fewer than 30 total connections (edges), with an average of 24.8 and standard deviation of 11.5. Only one of the 200 randomly selected networks has more than 50 connections. This network contains a ubiquitin protein, UBI4, which forms a hub that makes 43 of the 73 total connections in that network (S1 Fig). In summary, then, the 203 edges in our network make the connectivity in our network ~15.5 standard deviations from randomly selected networks of yeast proteins with a similar number of nodes. This corresponds to a *p*-value of 10^–54^.

### Molecular characteristics of the fat storage regulation network

The network contains extra- and intracellular energy/glucose detection mechanisms controlled by Mth1 and the AMP-activated protein kinase complex (AMPK) encoded by the *SNF1*, *SNF4*, and *SIP1* genes and their regulators *TOS3*, *ELM1*, and *SAK1*. High levels of extra- or intracellular glucose cause degradation of Mth1[29,30], while high AMP/ATP ratios cause activation of AMPK [31]. A reduction in signaling through either system is likely to produce a perceived surplus of energy that can stimulate fat storage. Mth1 and AMPK are connected to a set of transcriptional regulators and to four processes that may represent outputs. These are: 1) autophagy, which involves the TORC1 and vacuolar H+-ATPase (Vma) pathways [32]; 2) *de novo* fatty acid and sterol synthesis pathways; 3) MAP kinase (MAPK) pathways; 4) mRNA degradation, elongation, and initiation pathways involving the CCR4-NOT complex and its associated proteins. The MAP kinase pathways are involved in sugar and amino acid starvation responses, while the CCR4-NOT complex is known to repress the translation of mRNAs encoding proteins necessary for utilization of non-fermentable carbon sources and for glucose neogenesis [24,26].

We next examined if proteins that participate in the same process or pathway exhibit a higher tendency to have connections with each other than with other network proteins using a walk-trap algorithm. This function finds densely connected communities *via* random walks from one node to another [33]. The idea is that short random walks will tend to stay in the same community. This analysis showed that the network contains seven different communities (modules) composed of three or more proteins, and that each module is enriched for proteins that are involved in the same process (Fig 1C).

We performed an analysis of the Gene Ontology (GO) terms associated with each of the proteins in the network. GO includes three categories: Biological Process, Molecular Function, and Cellular Component. S3 Table shows a list of the GO terms that are enriched for network proteins relative to random yeast proteins, with *p*-values for the statistical significances of the enrichments. Many of these highly enriched categories are very general, with the top three being “growth”, “biological regulation”, and “protein phosphorylation”. Others overlap with the output processes described above (Fig 1), and are properties of the signaling pathways, biological processes, and cellular components represented in the network. Four of the modules within the network defined by the walk-trap algorithm (Fig 1C) are highly enriched for specific GO terms. These are terms related to MAPK signaling (green circles), vacuolar acidification and proton transport (dark blue circles), mRNA catabolic processes (light blue circles), and positive regulation of transcription (red circles). It has been previously shown that GO categories can correspond to clusters within the global yeast protein-protein interaction map[34].

The fact that proteins required for filamentous growth are overrepresented in the network (*p* = 1.8 x 10^–8^) is interesting, In limited nutritional conditions, yeast can adopt a filamentous growth pattern that permits a non-motile colony to explore its surroundings for additional nutrients. The induction of this state requires the action of the AMPK, MAP, and Tor kinase pathways [25], which form a considerable portion of the fat regulation network. We hypothesize that some yeast mutants that store excess fat do so because they are in a state of perceived energy excess, which is the opposite of the conditions in which filamentous growth would be favored.

In yeast, MAP kinase pathways are organized as cassettes composed of specific combinations of MAPKKKs, MAPKKs, and MAPKs, and these cassettes can cross-regulate one another [28]. Because we found mutations affecting multiple MAP kinase pathways, there may be redundancy between the cassettes with regard to control of fat storage. Consistent with this, we observed that a double mutant (*fus3 kss1*) lacking two MAPKs accumulates more fat than either single mutant (S2 Fig). We also note that mutations in TORC1 units and not TORC2 units cause an increase in fat storage, thus implicating only TORC1 in yeast fat storage regulation. This was confirmed by the fact that rapamycin (Rap), a selective inhibitor of the TORC1 complex[27], causes an increase in fat storage in wild-type yeast (S2 Fig).

We did not isolate any ‘lean’ mutants in our screen. This could be due to the fact that our growth media does not contain fatty acids that could be converted to phospholipids necessary for membrane synthesis during cell division, or, alternatively, to genetic redundancy. Indeed, in cases where a low fat storage yeast strain has been reported, a phenotype was only detected when more than one fatty acid synthesis gene is removed [35,36].

Most of the genes we identified have not been previously implicated in fat storage. Earlier screens were done for LD morphology defects, but only ~20% of the 171 genes previously reported to affect LDs showed an increase in fat storage in our assays [37–39]. This is not surprising, because these genes were identified in screens by altered LD morphologies in live mutant cells, not by measuring fat content. Because the goal of our screen was to find mutants with altered fat storage levels, not to study LD dynamics, we fixed cells in order to deactivate pumps that can affect dye uptake and block vesicular traffic to increase the specificity of the dyes to lipid droplets, and used quantitative assays to evaluate fat content [20,40,41].

### Mathematical analysis of network architecture

The fact that the interconnection density of the fat storage regulation network is much greater than that of any randomly generated network of yeast proteins (S1 Fig) implies that the majority of the connections within the network have biological relevance. This makes yeast fat regulation an ideal system in which to examine whether a biological network conforms to a mathematical model for network design. See S1 Text for detailed information on methods for mathematical analysis used in this paper.

Many real-world networks, such as the Internet, power grids, and social networks, have been studied, and they tend to have certain features in common. A social network, for example, contains many different local clusters of people that are linked to each other by mutual acquaintances, so that any person within the cluster can be reached from another person by a small number of steps. Social networks also contain celebrities (“hubs”), who have exponentially more followers than does an average member of the network. Similarly, biological signaling networks tend to contain modules and have hub-like proteins that make many more connections than other proteins.

These features of real-world networks have been simulated using graph theory, which is the study of systems of objects, referred to as nodes, and their relationships, referred to as edges or connections. The properties of networks generated by graph generation models can be characterized by a variety of different parameters (for review see [42]), including degree distribution *P(k)*, global clustering coefficient *C*_*g*_, modularity *M*, and path length *L*. *P(k)* is the probability that a given network node has a certain number of connections, while *C*_*g*_ is a measure of the degree to which nodes in a network tend to cluster together [43]. *M* is a measure of the division of a network into communities or modules, produced by the tendency of some nodes to form connections primarily within the community to which they belong. Finally, *L* is the average number of steps along the shortest paths that connect all possible pairs of network nodes.

**Fig 2:**
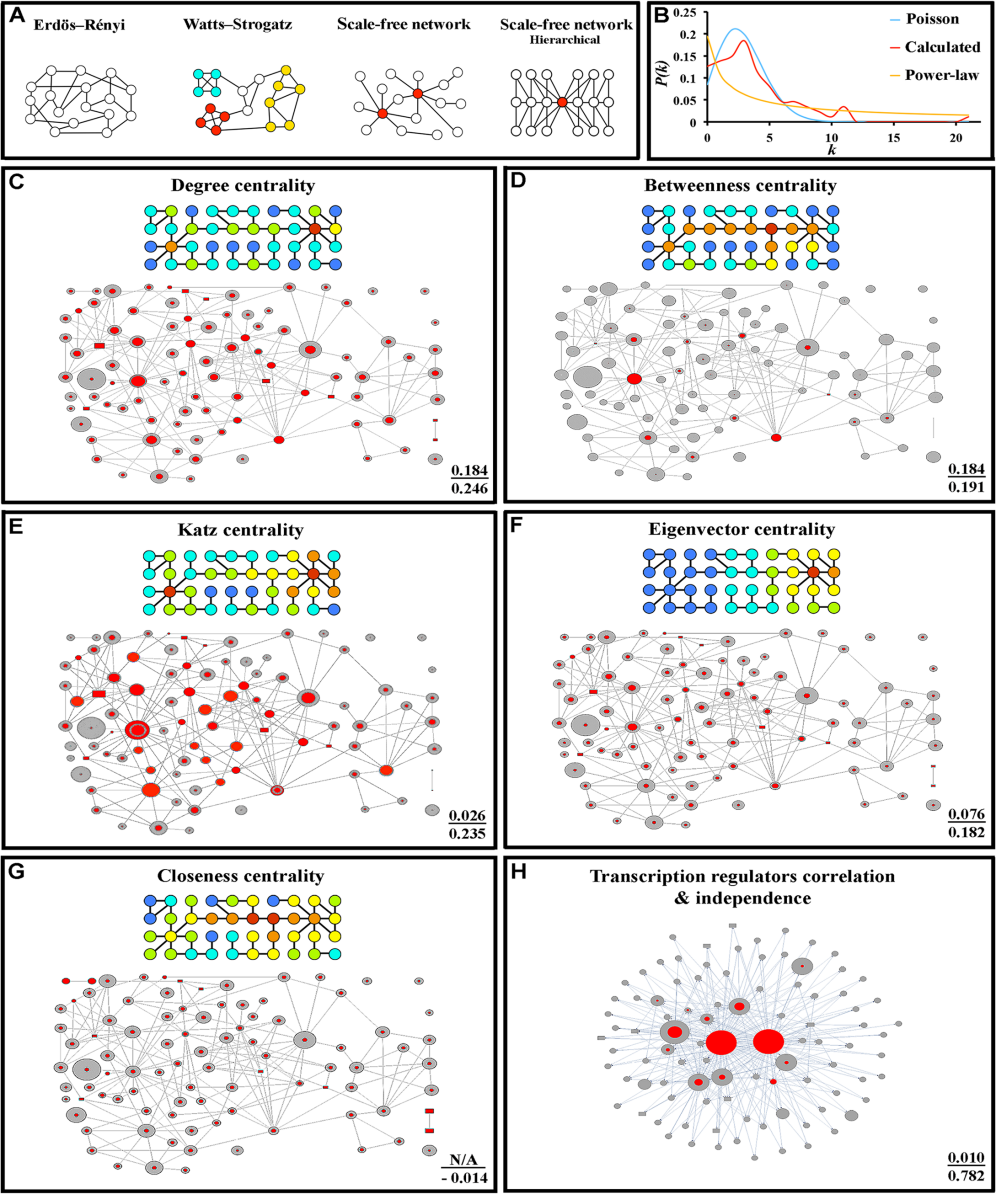
The fat storage regulation network has a Watts-Strogatz topology in which the importance of a protein is associated with Katz centrality. (A) Representations of the four major types of network topology. Nodes with the same color are members of the same module, while red circles in the scale-free network diagrams represent hubs. (B) The calculated *P(k)* distribution of the fat storage regulation network (red line) as compared to Poisson (blue line) and power-law distributions (yellow line). (C-H) The upper cartoon in each panel is a heat map of a small toy network showing the relative centrality for each node (high-low order: red-orange-yellow-green-aqua-blue). The lower diagram shows the experimental network map, with the sizes of the grey circles showing the relative impact on fat levels of mutation in the gene encoding that protein, and the sizes of the overlaid red circles represent the centrality values for each node. (H) is a map of transcriptional regulation, with the sizes of the grey circles indicating the number of genes regulated by a given transcription factor or global regulator. Upper black text: *p*-value for independence testing (Blomqvist β test). Lower black text: correlation.

The simplest model used to generate networks is the Erdos-Renyi model [44], characterized by an equal probability of forming connections between any two nodes (Fig 2A). Erdos-Renyi networks have a degree distribution similar to a Poisson distribution (Fig 2B), and have low *C*_*g*_, *M*, and *L* values. Most real-world networks are not approximated well by this model, because Erdos-Renyi graphs lack local clustering and hubs.

The Watts-Strogatz model was designed to produce better models of real-world networks by remedying the lack of clustering in Erdos-Renyi networks. Watts-Strogatz graphs are produced by randomly moving the edges of a regular ring lattice, thus producing “criss-cross” connections across the ring[19]. This does not imply that a real network approximated by the Watts-Strogatz model contains a structure resembling a ring lattice. Moving edges on a ring lattice is simply the algorithm by which these graphs are generated. Similarly, Erdos-Renyi and scale-free graphs (see below) are also generated by specific algorithms, but this does not imply that real networks with similar topologies are built using these algorithms. Watts-Strogatz networks are expected to have a degree distribution that depends on the rewiring probability β, which is related to the number of new connections between nodes that are introduced into the ring lattice. β varies between 0 (a regular ring lattice) and 1 (a lattice where so many connections are changed that it approximates an Erdos-Renyi graph). For moderate to high rewiring probabilities, the degree distribution of the network is a binomial distribution, which in the limit (β = 1) approximates a Poisson distribution. For a low rewiring probability (β near 0), the distribution resembles the *P(k)* of a regular ring lattice, which is a delta function. In all cases, graphs of *P(k)* as a function of *k* for such networks have a distinct peak. Networks generated by the Watts-Strogatz model have local clustering and small-world properties, with high *C*_*g*_ and *M* values relative to those of Erdos-Renyi networks[19] (Fig 2A).

Scale-free networks have a *P(k)* distribution that follows a power law, meaning that a few hub nodes make exponentially more connections than other nodes in the network. A graph of *P(k)* as a function of *k* for a scale-free network resembles an exponential decay curve (Fig 2B). The best-known model for generating such networks is the Barabási–Albert model, where the network evolves *via* the addition of new nodes that preferentially form connections to highly-connected nodes[45]. This model can account for the hubs found in real-world networks, but not for local clustering. Hierarchical scale-free networks are composed of modular units of nodes and connections that are combined in an iterative manner, while non-hierarchical scale-free networks have hubs but are not arranged in an organized pattern (Fig 2A). Some have argued that protein networks within cells are likely to be non-hierarchical scale-free networks[46], but others have found that real biological networks cannot be made to fit a power-law distribution[47,48].

### The fat storage regulation network resembles a Watts-Strogatz small-world network

The *P(k)* distribution of the experimental network has a distinct peak, and resembles a Poisson distribution more closely than an exponential decay curve, which indicates that the network is not scale-free (Fig 2B). To quantify this statement, we considered the SSE (standard sum of squares due to error) between a binomial distribution and the observed *P(k)* distribution of the experimental network. The minimal SSE obtained between a binomial distribution and the degree distribution for the experimental network is 0.012. The mean SSE on a selection of 200,000 simulated random (Erdos-Renyi) networks with the same expected distribution was 0.0099, with a standard deviation of 0.0057. This tells us that the SSE value of our network is less than half a standard deviation from the expected value. By contrast, the SSE obtained by fitting the experimental network to a power-law distribution was over two standard deviations from this expected mean. This provides strong evidence that the fat storage regulation network is much more likely to be approximated by an Erdos-Renyi model or a Wattz-Strogatz model than by a scale-free model (Fig 2B and S4 Table).

The experimental network has a relatively small *L* value (3.18), which is essentially the same as the expected *L* values for simulated random (Erdos-Renyi) networks (3.19). This does not distinguish between the Watts-Strogatz and Erdos-Renyi models, since both produce networks with low *L* values. However, the *C*_*g*_ and *M* values for the experimental network (0.22 and 0.567, respectively) are higher than the expected values for simulated Erdos-Renyi networks (0.048 and 0.44), but are in agreement with the Watts-Strogatz model for a moderate rewiring probability (β = 0.238). Its high *C*_*g*_ value and low *L* value characterize the experimental network as small-world. This small-world topology is a distinctive feature of the Watts-Strogatz model.

In a scale-free network, loss of peripheral nodes will have little effect on network parameters, but loss of hub nodes can produce major changes in path length *L*. An analogy to this is that shutdown or dysfunction of a hub airport (*e*.*g*., O’Hare in Chicago) due to bad weather will cause many travelers to have to take additional flights to reach their destinations. However, shutdown of a small regional airport will have no effect on the global pattern of air travel. To evaluate whether our network is vulnerable to deletion of any nodes, we determined the contribution made by each protein to the topological parameters of the entire network. This was done by calculating *L*, *C*_*g*_, and *M* for the network and then recalculating those parameters (*L*_*-P*_, *C*_*g-P*,_ and *M*_*-P*_) when each network protein and its connections were removed. Only minor changes to these values were observed in response to removal of any one protein (<10%; S2 Table). This is of interest, because it argues that the Watts-Strogatz topology of the network may contribute to the robustness of its topological parameters to deletion of any node. Unlike scale-free networks, it lacks essential hub nodes for which elimination of function by mutation or damage would dramatically alter the entire network. We also simultaneously removed all eight of the proteins encoded by essential genes that were added to the network (see above), to ensure that these proteins were not required for network properties. This produced no significant changes in *L* or *M*, and only a 10% decrease in *C*_*g*._

To evaluate the relationships between the experimentally determined fat storage regulation network and manually curated GO categories, we generated networks of protein-protein connections for the proteins in yeast Biological Process categories, since fat storage regulation is more like a Biological Process than it is like a Molecular Function or Cellular Component. These categories range in size from a few proteins to more than 250. We then calculated edge density (the number of edges/number of possible edges), *C*_*g*_, and *M* for these networks and compared these numbers to the values for the fat storage regulation network. S3 Fig shows that the edge density for our network is around the median for the GO categories. *C*_*g*_ is below the median, and *M* is above the median. These data indicate that the experimental fat storage regulation network has similar properties to those of networks formed from the proteins within GO Biological Process categories. Proteins in a ‘typical’ Biological Process category are extensively interconnected, and exhibit more clustering but less modularity than the proteins in the fat storage regulation network.

### The importance of a protein to the network’s function is related to its Katz centrality score

Because the fat regulation network was defined by a quantitative genetic screen, it has the useful property that the importance of a given protein to network output can be defined by the severity of the fat storage phenotype (the amount of fat added above wild-type levels) for a deletion mutation in the gene encoding that protein. This is graphically depicted in Figs 1 and 2, where the sizes of the circles in the network diagrams are proportional to the strength of the phenotype produced by loss of the corresponding protein. This allows us to evaluate the relationship between a node’s position in the network and its importance for network function. If such a relationship can be established, it can be used by future investigators to find the essential elements in other biological networks that are found to fit the Watts-Strogatz model, but for which quantitative information on phenotype is not available.

In network analysis, the centrality of a node, *C(v)*, refers to indicators that identify the most important nodes [49]. For example, centrality analysis has been used to find the most influential person in a social network. There are many types of centrality, each of which emphasizes a certain quantifiable attribute of a given node. We examined the five standard centrality types (Degree, Betweenness, Closeness, Eigenvector, and Katz centralities) [49–51] to measure these attributes. For degree centrality, the value is evaluated by counting the number of directly connected nodes. Betweenness centrality is evaluated by counting the number of shortest paths that pass through a given node. Closeness centrality is a sum of the inverses of the shortest path lengths. Eigenvector centrality is an implicitly defined measure evaluated by asserting that the centrality of a single node is proportional to the sum of the centralities of all nodes it is connected to. For Katz centrality, the importance of a node is determined by how many nodes it is path-connected to, with a penalty that increases exponentially with the path length between those nodes. The colored heat-map diagrams in Fig 2C–2G show the values of each type of centrality for each node in a small toy network, with red indicating the highest centrality. The diagrams below superimpose centrality values, indicated by the sizes of the red circles, on the grey circles that indicate phenotypic severity.

We assessed the relationships between the centrality of a given node and the severity of the fat storage defect exhibited when the gene corresponding to that node is removed, using both independence and correlation testing (Fig 2C–2H and S4 Table). Dependence is any statistical relationship between two random variables, while correlation is a special type of dependence that can be used to predict the magnitude of the change that will occur in one variable in response to changes in a linked variable. Two random variables that are independent necessarily have low correlation, but variables with low correlation can be dependent. For example, driving while intoxicated and fatal car accidents are clearly not independent of each other, but the correlation between the two is only moderate, since only 32% of such accidents involved an intoxicated driver and most intoxicated drivers do not have an accident. Dependence is a more general and arguably more useful measure, as it is able to detect nonlinear relationships, whereas correlation assumes a simple linear dependence.

The only centrality measure that passed five separate independence tests (Blomqvist β, Goodman-Kruskal, Hoeffding D, Kendall tau, and Spearman Rank) was Katz centrality (Fig 2E), for which the *p*-value (probability of independence) was <0.027 for all 5 tests. There are good reasons that Katz centrality gives the best dependence score, which are based on how the centrality measures are defined. First, a good measure of the importance of a protein node should be global, and not just depend on proteins that are directly connected to it. Second, those nodes related by longer paths should have a smaller effect on each other’s function than those related by shorter paths. These attributes are held by closeness, eigenvector and Katz centrality measures. The effect of adding a node on the closeness centrality depends on the reciprocal of path lengths, for eigenvector centrality it is loosely related to degrees of the node, and for Katz centrality, the effect will become exponentially smaller the as the path length increases. In many models, it is reasonable to assume that only a fraction of a signal passes through a node to each of the other nodes to which it connects, and this assumption predicts an exponential drop-off.

Although the statistical analysis shows a clear relationship between the severity of the fat storage phenotype and Katz centrality, the moderate correlation between these parameters (0.24) suggests that this is not necessarily a linear relationship. Fig 2E displays this graphically. It shows that many of the larger grey circles have corresponding large red circles, but there are some large grey circles with very small red circles, indicating low Katz centrality. This shows that the quantitative impact on fat levels caused by removal of a network protein cannot always be predicted from its Katz centrality score in a linear way. In particular, mutations eliminating transcriptional regulators that have low Katz centrality scores often have large impacts on fat levels (*e*.*g*., Spt10). This may be due to the fact that the importance of a transcriptional regulator to the network is more likely to be related to the number of gene targets whose expression it controls than to the number of proteomic connections it makes. Most of the transcription factors that regulate fat storage levels bind to the promoter regions of genes that are themselves part of the network, creating potential feedback loops [52] (Fig 2H). Ino2, Srb5, and Ctk1 bind to the promoter regions of up to 40% of network genes. In total, 67% of network genes have network transcription factors other than the global regulators Hhf1 and Spt10 that bind to their promoter regions. The number of network genes that a given network transcriptional regulator binds to is an excellent predictor (correlation = 0.787; probability of independence <0.01; S4 Table) for the severity of the fat storage defect when the gene that encodes it is removed.

### The network is divisible into subnetworks that regulate different aspects of fat metabolism

Another useful feature of yeast fat regulation is that mutants selected for increased fat content can also be examined for related phenotypes, such as LD morphology and metabolic alterations. We can then ask whether the genes and proteins that share these “sub-phenotypes” also form networks, and examine the relationships between these subnetworks and the larger fat regulation network.

**Fig 3:**
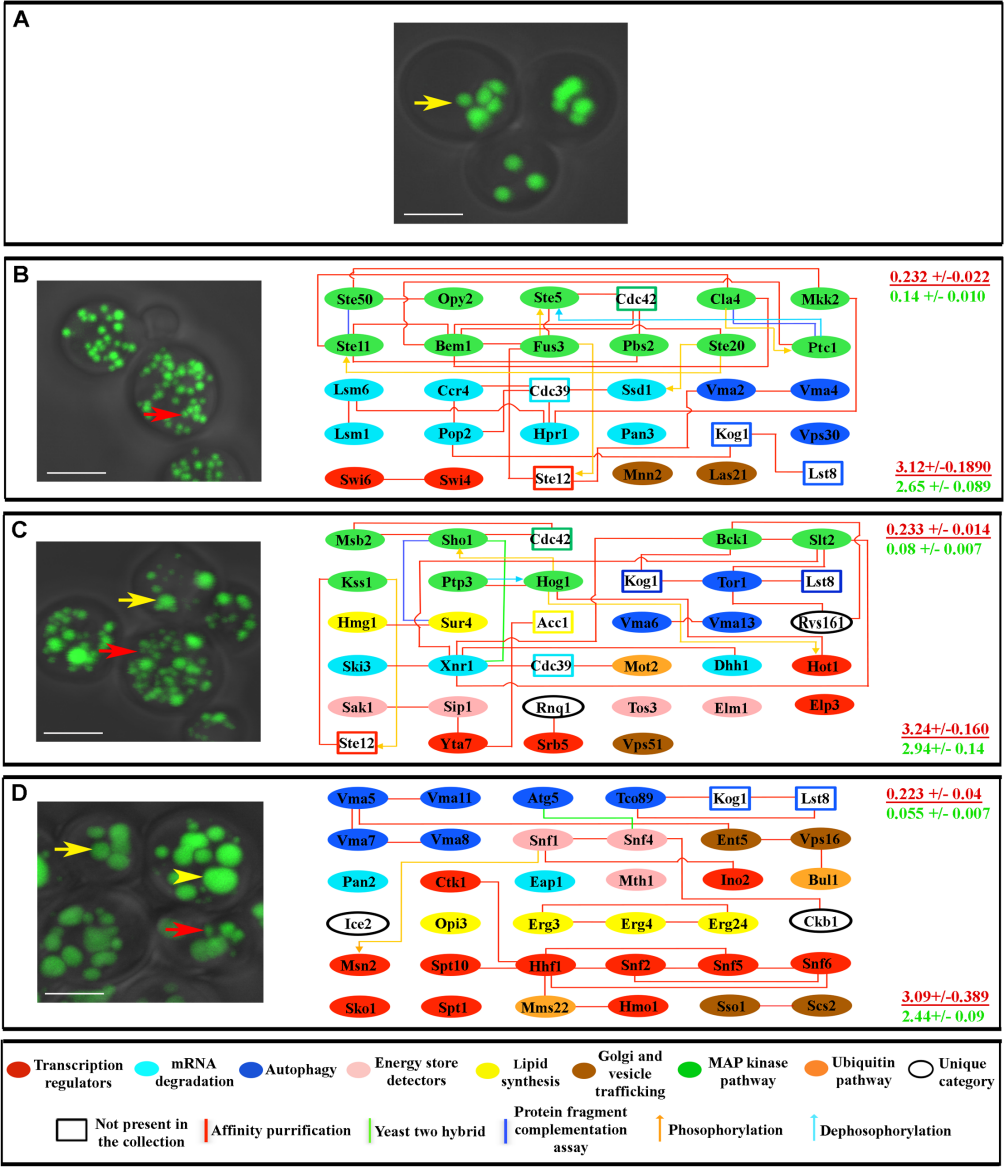
Genes for which mutations produce similar LD phenotypes affect connected proteins. (A) Fixed wild type yeast cells stained with BIODIPY 493/518, as visualized by confocal microscopy. Most cells have 3–8 LDs that are ~0.4 μm in diameter (yellow arrow). (B) Mutants with numerous and small LDs (red arrow), as exemplified by *fus3Δ* (left). The right sides of panels B-D show the genes for which mutants display this phenotype and their proteomic connections. (C) Mutants with a mixture of small and normal size LDs (red and yellow arrows, respectively), as exemplified by *mot2Δ* (left). (D) Mutants with supersized LDs, in addition to large and small LDs (yellow arrowhead, yellow arrow, and red arrow, respectively) as exemplified by *snf4Δ* (left). At the right side of each panel are indicated the global clustering coefficient (upper) and path length (lower) for each subnetwork (red font), presented over the mean of values from 10,000 generated simulated random networks with the same degree distribution and vertex count as the subnetwork in that panel (green font). Key for diagrams as in Fig 1. Scale bar, 4 μm.

Yeast LDs are composed of triglycerides and sterol esters surrounded by a monolayer of phospholipids. LDs may increase in size and/or number when trigylceride levels increase. A wild-type fixed yeast cell usually has three to eight LDs that are around 0.4 μm in diameter (Fig 3A). We examined all the mutants for LD morphology phenotypes, grouped them into three phenotypic classes, and created subnetworks encompassing all the proteins for each class. Class I contained mutants with small but numerous LDs, class II contained mutants with mixed populations of normal and small LDs, and class III contained mutants that have giant LDs (Fig 3). Each of the three subnetworks was highly interconnected, indicating that members of the same morphological class tend to have mutations affecting proteins that are connected to each other (S5 Table).

**Fig 4:**
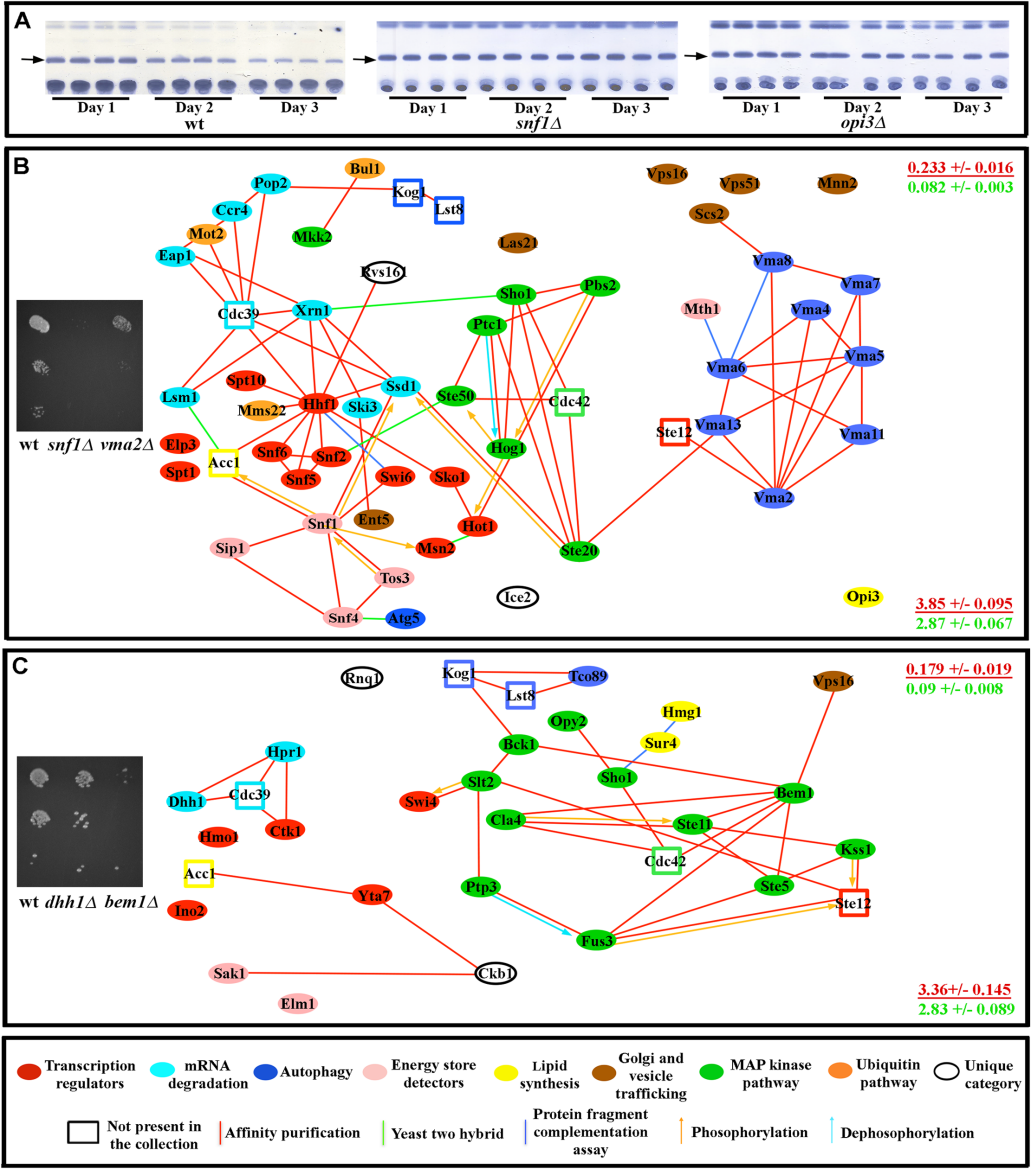
Genes for which mutations affect carbon source usage affect connected proteins. (A) TLC assays of wild-type yeast (four replicates each) show a gradual reduction in the intensity of the triglyceride band (arrows) as starvation continues for three days. This reduction is either not observed in mutants (e.g., *snf1Δ*) or occurs at a slower rate (e.g., *opi3Δ*). See Supplement for network analysis of fat store degradation. (B) Mutants that show slow or no growth on 3% glycerol, as exemplified by *snf1Δ* and *vma2Δ* mutants (image on left shows a dilution series from top to bottom). (C) Mutants that show slow or no growth on 0.1% lard and/or 0.1% palmitic acid, as exemplified by *dhh1Δ* and *bem1Δ* mutants (image on left). Mutations causing similar carbon source utilization defects affect genes that encode proteins that tend to be proteomically connected to one another (diagrams in (B) and (C). At the right side of each panel is the global clustering coefficient (upper) and path length (lower) for each subnetwork (red font), presented over the mean of values from 10,000 simulated random networks with the same degree distribution and vertex count as the subnetwork in that panel (green font). Key for diagrams as in Fig 1.

Fat levels are influenced by the rate of fat storage utilization, the rate of *de novo* fatty acid synthesis, and the level of caloric intake. We examined fat storage utilization by subjecting all of the mutants to glucose starvation for three days. ~85% of the mutants showed no reduction in fat storage or a reduced rate of fat storage depletion compared to wild-type when starved, indicating defects in the ability to use stored fat to meet energy demands (Fig 4A and S6 Table).

To be used as an energy source, fats have to be broken down to glycerol and fatty acids, which are used by mitochondria for ATP production [53,54]. We evaluated the overall status of mitochondria in all mutants with reduced fat utilization rate by growing them on glycerol. ~60% of mutants showed either slowed or no growth on glycerol, suggesting defects in mitochondrial function. All of these mutants also failed to grow on palmitic acid and lard. Another 26 mutants did grow on glycerol, but failed to grow on either palmitic acid or lard or both. Mutations that produce a similar growth defect on a given carbon energy source tend to affect proteins that are connected to one another, forming subnetworks (S6 Table and Fig 4B and 4C). These results show that a significant portion of the network is dedicated to maintaining normal mitochondrial function. Indeed, nearly half of the genes we identified were previously implicated in mitochondrial function[53].

**Fig 5:**
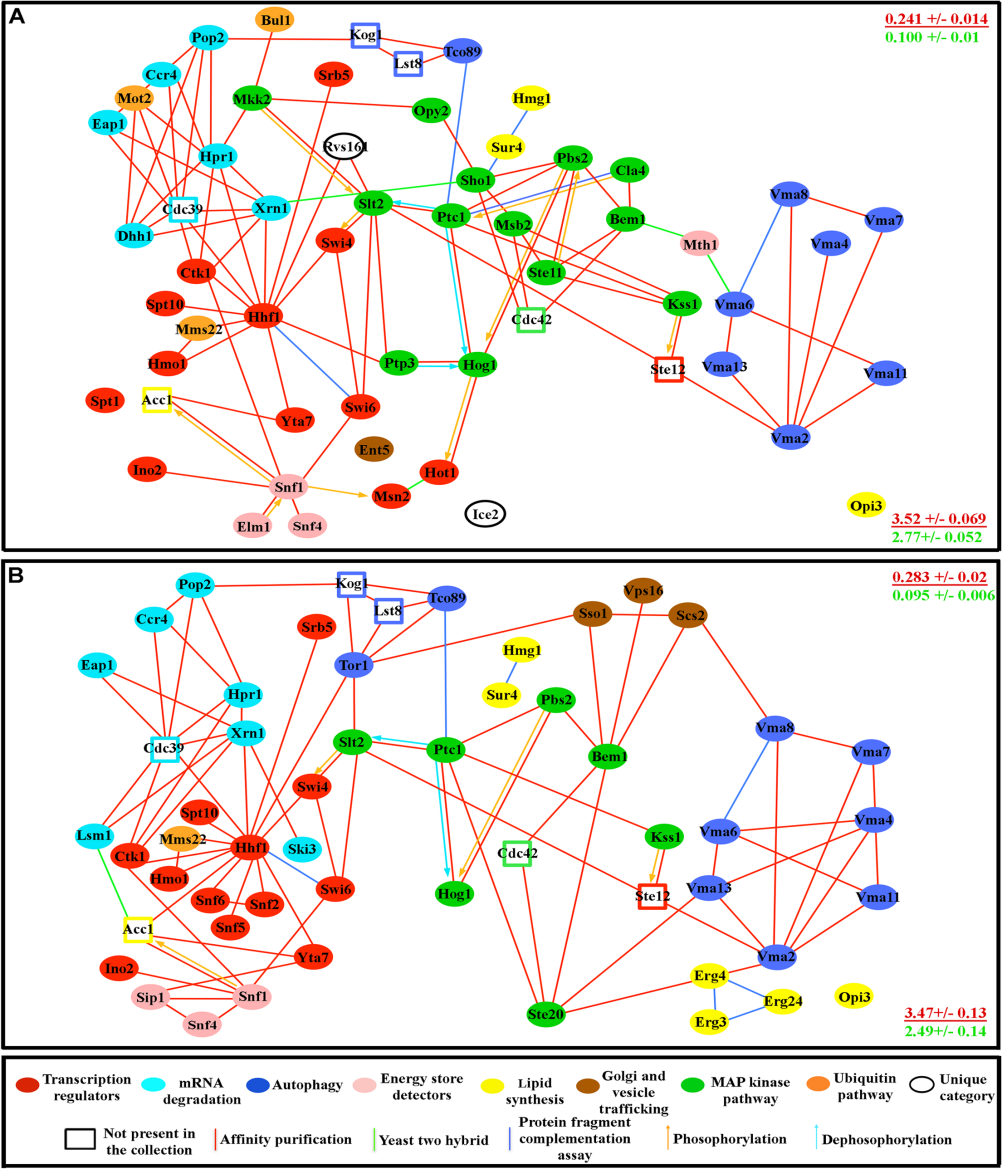
Genes for which mutations affect conversion of glucose or aspartic acid to fat affect connected proteins. (A) Proteins encoded by genes for which mutation produces more than a 20% increase in the rate of conversion of ^14^C labeled D-glucose to fat relative to wild-type tend to be proteomically connected to one another. (B) Proteins encoded by genes for which mutation produces more than a 20% increase in the rate of conversion of ^14^C labeled L-aspartic acid to fat relative to wild-type tend to be proteomically connected to one another. At the right side of each panel is the global clustering coefficient (upper) and path length (lower) for each subnetwork (red font), presented over the mean of values from 10,000 simulated random networks with the same degree distribution and vertex count as the subnetwork in that panel (green font). Key for diagrams as in Fig 1.

In our growth conditions, cells were provided with glucose and a mixture of amino acids that can be used for *de novo* fatty acid synthesis. To examine if any of the mutants have alterations in this process, we grew them on media containing either ^14^C-labeled D-glucose or ^14^C-labeled L-aspartic acid. About 30% of the mutants showed an increase in the conversion of either D-glucose or L-aspartic acid to fat. Mutants that exhibited increased conversion of either nutrient to fat tend to encode proteins that are part of a connected subnetwork (Fig 5 and S7 Table).

To test the hypothesis that genes for which mutants exhibit similar physiological profiles would encode proteins that formed subnetworks, we examined all of the potential subnetworks encoded by subsets of genes with similar detailed sub-phenotypes, in the same way that we had examined the complete network. We found that these subnetworks also exhibited Watts-Strogatz small-world topology, with relatively high clustering coefficients (0.179–0.28) and short path lengths (3.09–3.85). To determine if the subnetworks represented functional subsets of the larger network, we then compared each subnetwork to randomized simulations of 10^4^ networks with identical degree distribution and vertex counts. In all cases, the experimentally observed subnetworks had much higher clustering coefficients than their simulated equivalents (red and green numbers on the right sides of the panels of S5–S7 Tables and Figs 3–5), indicating that the subnetworks were selected by evolution, and do not represent randomly chosen subsets of the complete network. We also observed that in all cases, these subnetworks are not confined to a single module, but span modular boundaries. These results suggest that the larger network that controls fat storage contains within it smaller networks that govern different aspects of physiology related to a cell’s decision whether to store fat or metabolize it for energy.

### Patterns of communication among distant network nodes are consistent with the Watts-Strogatz small-world topology

Having established that the fat regulation network fits the Watts-Strogatz model, we then conducted experiments in which we perturbed the functions of multiple network nodes and measured the effects of these perturbations on fat content. This allowed us to evaluate whether the patterns of communication among distant nodes in the real network are consistent with the Watts-Strogatz topology. We call these patterns “signal propagation”, because, although they are not measured dynamically, they reflect the movement of information through the network. They thus represent signal flow along signal transduction pathways and crosstalk between these pathways.

We conducted “chemogenomic” experiments in which we simultaneously perturbed the functions of multiple network nodes by treating yeast bearing a deletion mutation in each network gene with a set of drugs that block the functions of specific network pathways, followed by measuring the effects of these perturbations on fat content. The approach of adding drugs to single mutants to block multiple nodes was chosen in order to avoid the slow growth or lethality frequently observed for double mutants (see [55]), as well as the necessity to construct all possible double mutants. The chemogenomic approach has often been employed in the yeast system to map synergistic and antagonistic relationships between drug targets and other genes (for recent reviews see [56,57]).

**Fig 6:**
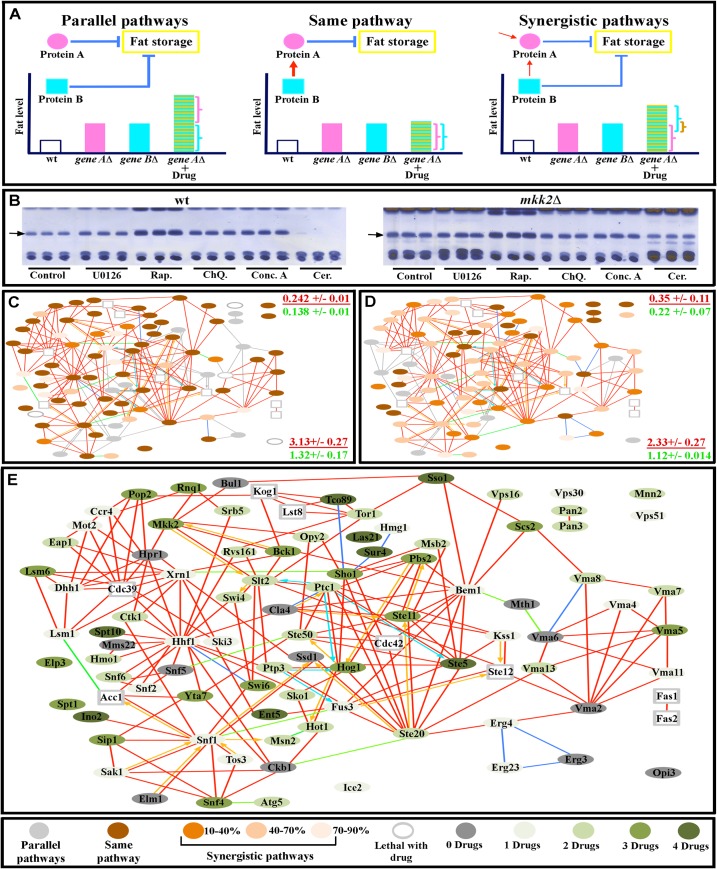
Internode communication patterns deduced from drug-mutant interactions. (A) Representations of signaling relationships between drug targets and mutant genes. In the diagrams, gene A encodes protein A, while gene B encodes protein B, which is the target of the drug. (B) TLC assays of wild-type (wt) yeast (three replicates each) showing the impact of different drug treatments on triglyceride bands (arrows). All mutants were subjected to the same treatments and analyzed in the same manner, as exemplified by the three replicas of *mkk2∆* for each drug (note that U0126, which targets MAPKKs, does not increase fat levesl in *mkk2∆* cells relative to untreated mutant cells). (C-D) Networks of interactions between mutants and U0126 (C) and Rap (D). Protein names are given in the corresponding larger diagram in (E). Note that the majority of proteins have signaling interactions with the drugs that range from “same pathway” (no enhancement of drug effect by mutation; dark brown), to different degrees of synergism (enhancement of drug effect by mutation, indicated by different shades of light brown), and there are fewer cases of parallel pathway (independent) relationships, in which the drug and mutant effects are additive (light grey). These parallel pathway relationships are like the synthetic negative interactions seen in double mutant studies. Corresponding data for ChQ., Conc. A, and Cer. are in S4 Fig. At the right side of panels (C) and (D) are indicated the global clustering coefficient (upper) and path length (lower) for a subnetwork of all proteins having a “same pathway” relationship to that drug (red font), presented over the mean of values from 10,000 simulated random networks with the same degree distribution and vertex count (green font). (E) Diagram of same pathway signaling relationships between drugs and network proteins. The circle color represents the number of drugs with which a protein has a same pathway relationship.

We selected five drugs that blocked signal propagation through specific network pathways. The first drug was U0126, an inhibitor of mammalian MAPKKs [58] that has been shown to block reporter expression controlled by the MAPK mating factor response pathway [59]. The second drug was Rap, an inhibitor of the mammalian and yeast TORC1 complex[27]. The third and fourth drugs were chloroquine (ChQ) and concanamycin A (Conc. A); these are both known to block the acidification of vacuoles by the Vma pump [60–63]. The fifth drug was cerulenin, which inhibits both yeast and mammalian fatty acid synthase [64,65] and thereby eliminates fat synthesis; cerulenin also served as a control to ensure that the effects of the mutations were dependent on network output. The specificity of the drugs we chose was confirmed by the fact that they did not produce increases in fat levels in mutants missing their potential targets (since in those mutants signals from the drug targets are already absent), and by the observation that they produce fat levels similar to mutations that remove these targets (see Supplemental Materials and Methods). Regardless of whether the drugs were completely specific for particular targets, they clearly caused perturbations in network function, as indicated by their ability to alter fat levels in wild type yeast (Fig 6B). The effects of these perturbations on mutant networks, each of which lacks a single node, can therefore be used to analyze internode communication within the network.

We defined three types of signaling relationships between network proteins that are removed by mutation and those whose activities are blocked by a given drug. First, the proteins could be in independent signaling pathways. In such cases, the application of the drug would produce an additive effect, such that the amount of additional fat would equal the sum of the amount of fat added by the mutation and the amount added to wild-type yeast in response to drug treatment. This type of interaction is like the negative interactions between deletion mutations (or between deletion mutations and drugs) that are often observed in double mutant or chemogenomic analyses of yeast growth phenotypes (reviewed by[56]). The strongest form of this type of negative genetic interaction is synthetic lethality, in which double mutant cells (or mutant cells treated with drug) die, while single mutants or drug-treated wild-type cells are viable. Second, the proteins could be part of the same pathway. In such cases, treating the mutant with drug would produce fat levels that are no higher than those in wild-type yeast treated with drug, since pathway signaling had already been eliminated by the mutation. Third, the proteins affected by the drug and by the mutation could be components of two pathways that relay part, but not all, of their signals through each other. We refer to these as synergistic pathways. In such cases, addition of a drug affecting pathway 1 to a mutant affecting pathway 2 would produce an increase in fat levels that is less than the sum of the amount of fat added by the mutant and the amount added by the drug, since the portion of the signal relayed through the action of both pathways was already blocked by the mutation, and the drug could therefore only affect the fraction of the signal that was still active and available for inhibition. In other words, part of the fat level increase observed for the mutation alone would be due to partial blockage of the drug-affected pathway (Fig 6A).

During the course of our analyses, we noticed that none of the drug-mutant combinations produced fat levels that approach the levels of our “fattest” mutant, *spt10∆*, indicating that our analysis was not limited by cells reaching their maximum fat storage capacity. We also never observed a situation in which the amount of fat in given drug-mutant combination was greater than the sum of the amount added by the drug alone and the mutation alone. These observations indicated that the behavior of the network could be understood using our method.

First, we found that, for each of the four different drugs that increase fat, 12–15% of network proteins behave as if they are in pathways that are independent of the pathway affected by the drug (*e*.*g*., they have strong synthetic negative interactions with the drug target). Second, 58% of network proteins had either “same pathway” or “synergistic pathway” relationships with all four drug target pathways (S8 Table and Figs 6 and S4), indicating that there is extensive communication across the network and that network outputs reflect integration among multiple signaling pathways. The fact that proteins involved in such relationships can be in different regions of the network is consistent with the idea that the short path length characteristic of small-world networks facilitates signal propagation between distant parts of the network.

Third, proteins that had a “same pathway”-type relationship with a given drug target sometimes were in different network communities from the drug target. Some of these relationships suggested hitherto unknown interactions between pathways. For example, components of the CCR4-NOT complex involved in mRNA processing had “same pathway” type relationships to the MAPK node affected by U0126, showing that the output of CCR4-NOT was relevant to regulation of fat by the MAPK pathway (Fig 6D). Proteins with “same pathway” type relationships to a given drug could be grouped into subnetworks that had *Cg* values that were much higher than those of simulated random networks of the same size and connectivity (0.22–0.35), indicating that they have been selected by evolution. These subnetworks did show significant overlap with one another (sharing some of the same proteins), but each one had a unique combination of proteins that was specific for a given drug (S9 Table and Figs 6 and S4).

Fourth, there is a “sub-additive” response (that is, an increase in fat content that is less than the sum of the increase produced by the drug and that produced by the mutation) to loss of function of two nodes for most node pairs. Fifth, eight proteins had a “same-pathway” type relationship with all four drugs, qualifying them as points of convergence for network signals (Fig 6E). This might suggest that these are hub proteins within a scale-free network. However, the hypothetical power-law distribution shown in Fig 2B predicts that in order for our network to be truly scale-free, the number of hubs should not have exceeded two. Furthermore, of these eight proteins, only Ste5 had more than four connections, and hubs should make many more connections than other proteins in the network.

We compared our results to some recent analyses of double mutant and drug-mutant growth and metabolism phenotypes[55,66,67]. These are genome-wide studies, so only a small percentage of mutants (~5%) exhibited a double mutant interaction or an interaction with a given drug. In our study, we investigated a subset of genes that we had defined as critical for fat storage regulation, and examined interactions with drugs that we had selected due to their ability to affect fat content, so we observed that most mutants displayed interactions with all of the drugs. In the genome-wide studies, the frequency of synthetic (negative) interactions was much higher for genes within the same GO Biological Process category than for genes in different categories. For most categories, this frequency was between 10 and 18%[55], which is in the same range as the frequency of genes within the fat regulation network that have strong negative interactions with a given drug target (12–15%; “independent pathways” category). Thus, the experimentally defined fat storage regulation network, which has interconnection density and modularity values that are similar to those of some Biological Process categories (S3 Fig), also behaves somewhat like a Biological Process category with respect to chemogenomic interactions. Finally, about 1/3 of interactions observed in genome-wide studies were positive, but we did not detect any positive interactions. This is due to the fact that we only isolated genes for which mutation increases fat contes, so all of our phenotypes have the same sign. Cellular signaling pathways, however, contain both positive and negative regulators, and mutation of regulators with opposite sign can produce phenotypes of opposite sign.

We also compared the results of Rap treatment of fat storage mutants with a study that examined interactions between Rap and all nonessential genes for a different TORC1-dependent phenotype, expression of a DAL80 reporter that is induced by TORC1 inactivation by Rap or starvation[68]. Of the 63 Rap-specific genes they identified, only 6 (Bck1, Las21, Slt2, Srb5, Swi4, Swi6) were identified in our screen as mutations that increase fat content, suggesting that fat storage regulation and DAL80 induction are not closely related processes. However, two of these common genes (Srb5 and Swi6), increase induction of the DAL80 reporter by Rap[68] and also synergize with Rap to increase fat content (Fig 6 and S8 Table).

## Discussion

By screening the yeast (*Saccharomyces cerevisiae*) viable deletion collection for mutations affecting fat content, we discovered a densely interconnected network of 94 proteins that regulates fat storage (Fig 1). From a computational analysis of this network, we derived three major conclusions. First, the network is not scale-free, and can be best approximated by the Watts-Strogatz model, which has not been previously applied to biological signaling networks (Fig 2). This model can account for the high degree of clustering observed in the experimental network. Second, the importance of an individual protein to network function is dependent on a particular measure of centrality, Katz centrality, which is influenced by the topological relationships between the test protein and the other proteins within its network community or module (Fig 2). Physiological analysis showed that the fat regulation network is divisible into connected subnetworks which span community boundaries and affect specific aspects of lipid metabolism (Figs 3–5). Finally, by combining drug perturbations with genetics (chemogenomics), we showed that there is extensive cross-talk between the different signaling pathways represented in the network (Fig 6).

The small-world topology endows the network with useful features. Its short path length property allows distant nodes to communicate with each other through a small number of steps. Its topological parameters are robust with respect to removal of any one protein. In scale-free networks, the removal of highly connected hubs will cause substantial changes in network topology and may fragment them into unconnected subnetworks [69–71], while the absence of modular structure in the Erdos-Renyi model makes it inconsistent with the formation of biological networks that must receive different types of inputs and couple them to unique outputs.

Our results also show that the importance of a protein to network function is dependent on its Katz centrality score (Fig 2). The Katz centrality of a node is determined by the number of shortest paths that pass through it to all other nodes in the network, with penalties assigned to connections to distant nodes (nodes that are connected to the node of interest only through a proximal node). The reason that this type of centrality gives the best prediction of a protein’s importance to the function of the fat regulation network may be due to the fact that it combines the local attributes of a node within its community with the position of that community within the network as a whole. As such, it is suited to modular/community based networks, and we predict that future analysis of other such networks will reveal that some of the most important proteins will be identifiable by their Katz centrality scores. In the fat regulation network, the importance of some of the proteins with high Katz centrality can be explained based on their known roles in the module to which they belong. Two examples are Bem1, which is a MAP kinase pathway scaffolding protein [72,73], and Snf1, which is the catalytic subunit of the AMPK complex [29,31].

By analyzing other phenotypes associated with alterations in fat storage, such as LD morphology and utilization of carbon sources, we further demonstrate that the network can be divided into subnetworks that span molecular categories and modules, but affect specific aspects of lipid metabolism (Figs 3–5). All subnetworks contain proteins that have connections that span modular boundaries.

To examine whether the topological features described above are associated with biologically relevant properties, we examined signal propagation within the network by combining genetics and pharmacology (Fig 6). The results indicate that the network has a sub-additive response to perturbation, because the blockage of two pathways or proteins by a drug and a mutation usually produces effects that are smaller than the sum of those caused by the drug and the mutation individually. Of course, the network is not immune to alteration; the removal of a protein from the network does produce an increase in fat levels, since that is how the network was defined. Nevertheless, the attenuated response of the network to blockage of multiple nodes is consistent with the idea that network structure buffers it against external or internal perturbations. The fact that proteins in distant parts of the network often interact with each other, as indicated by the drug-mutant experiments, are in agreement with the short path length and small-world properties of the Watts-Strogatz model.

Unlike Erdos-Renyi networks, Watts-Strogatz networks are modular. In neural networks, modularity is known to facilitate multifunctionality, which can divide large tasks into smaller compartmentalized subtasks that can be executed efficiently [74–76]. Multifunctionality also allows neural networks to integrate different inputs and generate diverse output responses. The yeast fat regulation network has properties that are analogous to multifunctionality in modular neural networks, because it takes in multiple inputs, processes them, and generates multiple outputs in response. The integrated input of the network represents an evaluation of the available nutrients and energy stores. It is provided by the glucose level detection function of Mth1 and the AMP and starvation detection mechanisms of MAP kinase and AMPK pathways. The network then generates anabolic and catabolic outputs that are appropriate to those inputs. Our analysis of mutant phenotypes indicates that these outputs affect many aspects of cell physiology, such as LD morphology, mitochondrial function, and fat store utilization.

Biological signaling networks often contain hub-like elements, and some researchers have proposed that biological networks with hubs are best described by scale-free models[46]. However, real interaction network datasets that have been tested do not fit power-law *P(k)* distributions, which are diagnostic of scale-free networks [47,48]. The *P(k)* distributions of proteins in yeast proteomic networks generated by the two-hybrid method have been subjected to mathematical analysis, and a variety of models were tested, including Poisson and binomial distributions (characteristic of Erdos-Renyi and Watts-Strogatz networks) and power-law distributions (characteristic of scale-free networks) were tested. None of the models could be definitively proven or ruled out [18,77].

Almost all of the proteomic connections used to define the fat storage regulation network were identified by affinity purification/coprecipitation methods, which provide a reliable means to identify abundant and stable protein complexes that exist *in vivo* [7,8,10,12,78,79]. Our computational analysis clearly shows that this network’s topology is best approximated by the Watts-Strogatz small-world model (Fig 2B). This topology has significant regulatory advantages that are correlated with biologically relevant features, and we suggest that mathematical analysis of other proteomic networks defined by a combination of genetics and affinity purification methods may reveal that many signaling networks within cells are of this type.

## Methods

### Genetics and fat assays

A collection of haploid *MATa* nonessential yeast deletion strains was purchased from Thermo Scientific. The strains were transferred into 150 μl YPD media in a 96 well-plate and grown for three days at 30°C. 5 μl of the resulting culture was respectively transferred into 150 μl synthetic complete dextrose media [80] in a 96 well assay plates (black wall with transparent bottom) and grown for two days at 30° C. After the growth period, formaldehyde was added to each well to a final concentration of 4%, followed by incubation at room temperature for 20 minutes. The plates were then spun down at 3000 rpm for two minutes and the supernatant was discarded. The pellets were then resuspended in 150 μl PBS containing 0.125 μg/ml Nile Red and 0.003 μM DAPI, followed by incubation at room temperature for 20 minutes. Fluorescence was measured using a spectrophotometer for both Nile Red (Ex485/Em590) and DAPI (Ex 358/Em440). Results were plotted as a ratio between the Nile Red and DAPI signals. Any positive lines were regrown in eight replicas, of which 4 were stained as above; the others were processed in the same manner, except that Nile Red and DAPI were not added. The latter group was used to measure autofluorescence, the value of which was later subtracted from the final reading. Only lines that remained positive were taken to the second round of selection.

Lines that passed both the 96-well plate assay and the histological examination were subjected to the TLC assay. In this assay cultures were grown in complete synthetic 2% dextrose media for two days at 30° C, and 20 ml normalized culture of OD_600_ = 1 was obtained. Protein from 1ml of OD_600_ = 1 culture was extracted and measured and reading was used for additional normalization step, and we rarely observe a situation in which cultures with the same OD_600_ ending up having a different protein measurements. The twice-normalized culture was then spun down, and the resulting pellets were suspended in 200 μl 2:1 chloroform: methanol mixture, and three glass beads (2 mm) were added to each tube. The samples were subjected to continuous agitation for one hour, and vortexed three times (1 minute each) during that period. The samples were then spun down for 2 min. at maximum speed and the lower phase was isolated and transferred to a fresh tube. The isolated lower phase was spun down again and run on a TLC plate as described [20].

For confocal microscopy, yeast were grown on synthetic complete 2% dextrose media at 30° C for two days. The samples were then spun down and processed for microscopy [81].

For additional information see S1 Text (Supplementary Materials and Methods), which contains methods for starvation studies, growth on different carbon sources, drug treatment, and mathematical analysis.

## Supporting Information

S1 Fig Histogram of the number of edges in N = 200 randomly sampled subsets of the yeast proteome. To test whether or not the interconnection density of the fat storage network is significant, we compared its statistics to those of networks of comparable size chosen by random selection from the yeast proteome. These random sub-networks all had far less connectivity (average 24.8 edges, standard deviation 11.5) than the fat storage network (203 total edges). Almost all of these randomly sampled networks lacked sufficient connectivity to calculate meaningful network statistics such as shortest path length and global clustering coefficient.
(TIF)

S2 Fig Synergy between the Fus3 and Kss1 MAPKs, and the effects of TORC1 mutations and rapamycin in yeast. (A) *fus3*,*kss1* double mutants have a higher fat levels than either single mutant. S is 4 μg lard standard, black arrow is triglyceride band, 4 μl volume was used for each sample lane. (B) Mutations in genes encoding TORC1 components (*tor1* and *tco89)* cause an increase in fat storage levels (upper panel), while mutations in genes encoding TORC2 components (*avo2* and *bit61*) do not (middle panel). Treatment of wild type yeast with the TORC1 inhibitor rapamycin causes an increase in fat storage as compared to vehicle only control (lower panel). S is 4 μg lard standard, black arrow is triglyceride band, 4 μl volume was used for each sample lane.
(TIF)

S3 Fig Histograms of the relationships between fat storage regulation network and all yeast GO Biological Process categories. The four histograms show edge density (# edges/# possible edges), cluster coefficient (C_g_), modularity (M), and the number of nodes (proteins) for each GO Biological Process category. In each histogram the value of the parameter for the experimental fat storage regulation network is shown by a vertical red line.
(JPG)

S4 Fig Internode communication deduced from drug-mutant interactions. (A) Representations of signaling relationships between drug targets and mutant genes. Networks of interactions between mutants and ChQ, Con. A, and Cer. Protein names are given in larger diagram in Fig 6E. Note that the majority of proteins have signaling interactions with the drugs that range from “same pathway” (no enhancement of drug effect by mutation; dark brown), to different degrees of synergism (enhancement of drug effect by mutation, indicated by different shades of light brown), and there are only a few cases of non-interacting parallel (independent) relationships, in which the drug and mutant effects are additive (light grey). At the right side of panels are indicated the global clustering coefficient (upper) and path length (lower) for a subnetwork of all proteins having a same pathway relationship to that drug (red font), presented over the mean of values from 10,000 generated simulated random networks with the same degree distribution and vertex count (green font).
(TIF)

S1 Text Supplementary materials and methods. (DOCX)

S1 Table TLC quantification of fat levels in yeast mutants. (XLSX)

S2 Table Connection matrix of nodes and edges in the network. (XLSX)

S3 Table GO term classifications that are significantly enriched for network proteins. (XLS)

S4 Table Mathematical analysis and computational modeling of the different network topological parameters shown in Fig 2. (XLSX)

S5 Table Mathematical analysis and computational modeling of the LD morphology subnetworks shown in Fig 3. (XLSX)

S6 Table Mathematical analysis and computational modeling of the carbon source utilization subnetworks shown in Fig 4. (XLSX)

S7 Table Mathematical analysis and computational modeling of subnetworks of proteins for which mutations affect conversion of glucose or aspartic acid to fat as shown in Fig 5. (XLSX)

S8 Table Quantification of the increase in fat levels produced by drugs in mutants vs. wild-type, as shown in Fig 6. (XLSX)

S9 Table Mathematical analysis and computational modeling of the “same pathway” subnetworks in Fig 6. (XLSX)
