# Supplementary figures and images for: Experimental and Computational Analysis of a Large Protein Network That Controls Fat Storage Reveals the Design Principles of a Signaling Network
Source: PLoS Comput Biol. 2015 May 28;11(5):e1004264. doi: 10.1371/journal.pcbi.1004264 (PMC4447291; doi:10.1371/journal.pcbi.1004264)

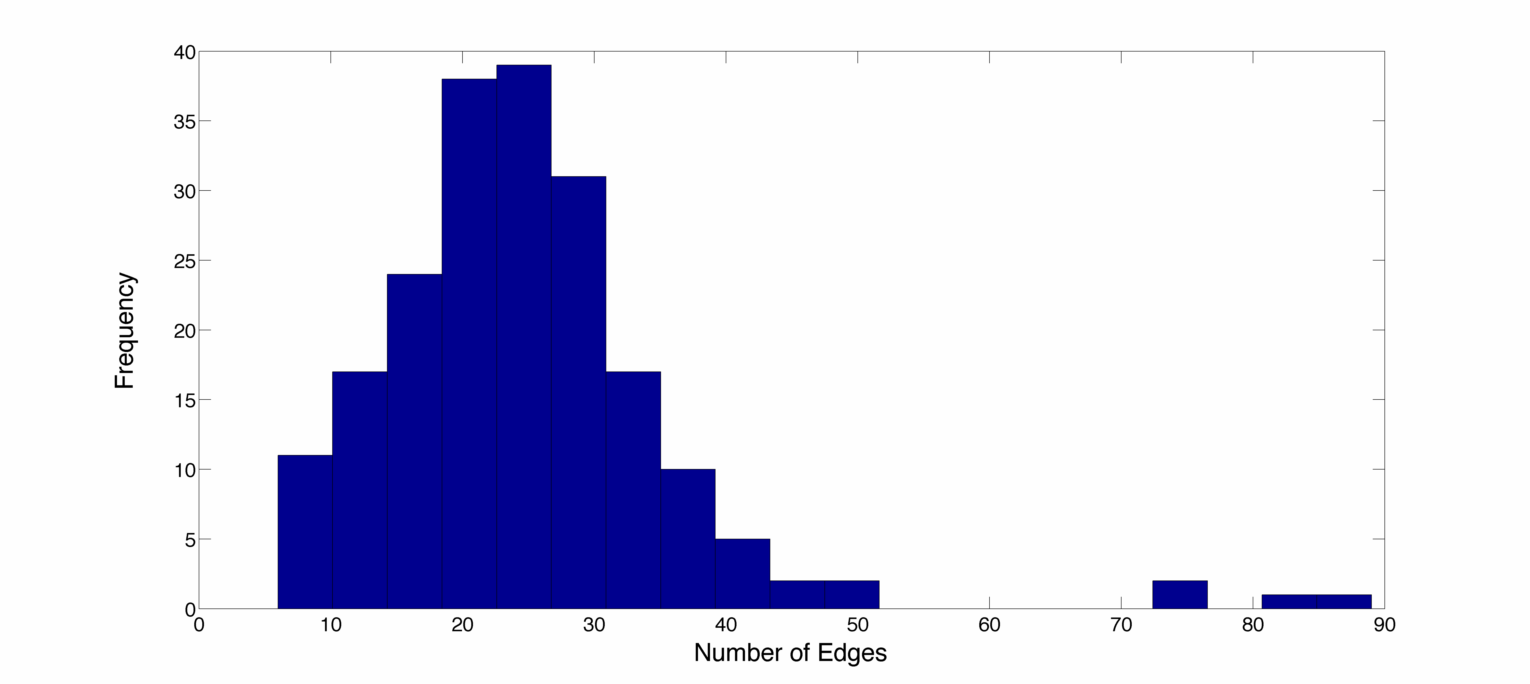

Supplement: S1 Fig — To test whether or not the interconnection density of the fat storage network is significant, we compared its statistics to those of networks of comparable size chosen by random selection from the yeast proteome. These random sub-networks all had far less connectivity (average 24.8 edges, standard deviation 11.5) than the fat storage network (203 total edges). Almost all of these randomly sampled networks lacked sufficient connectivity to calculate meaningful network statistics such as shortest path length and global clustering coefficient. (TIF) [file pcbi.1004264.s001.tif]

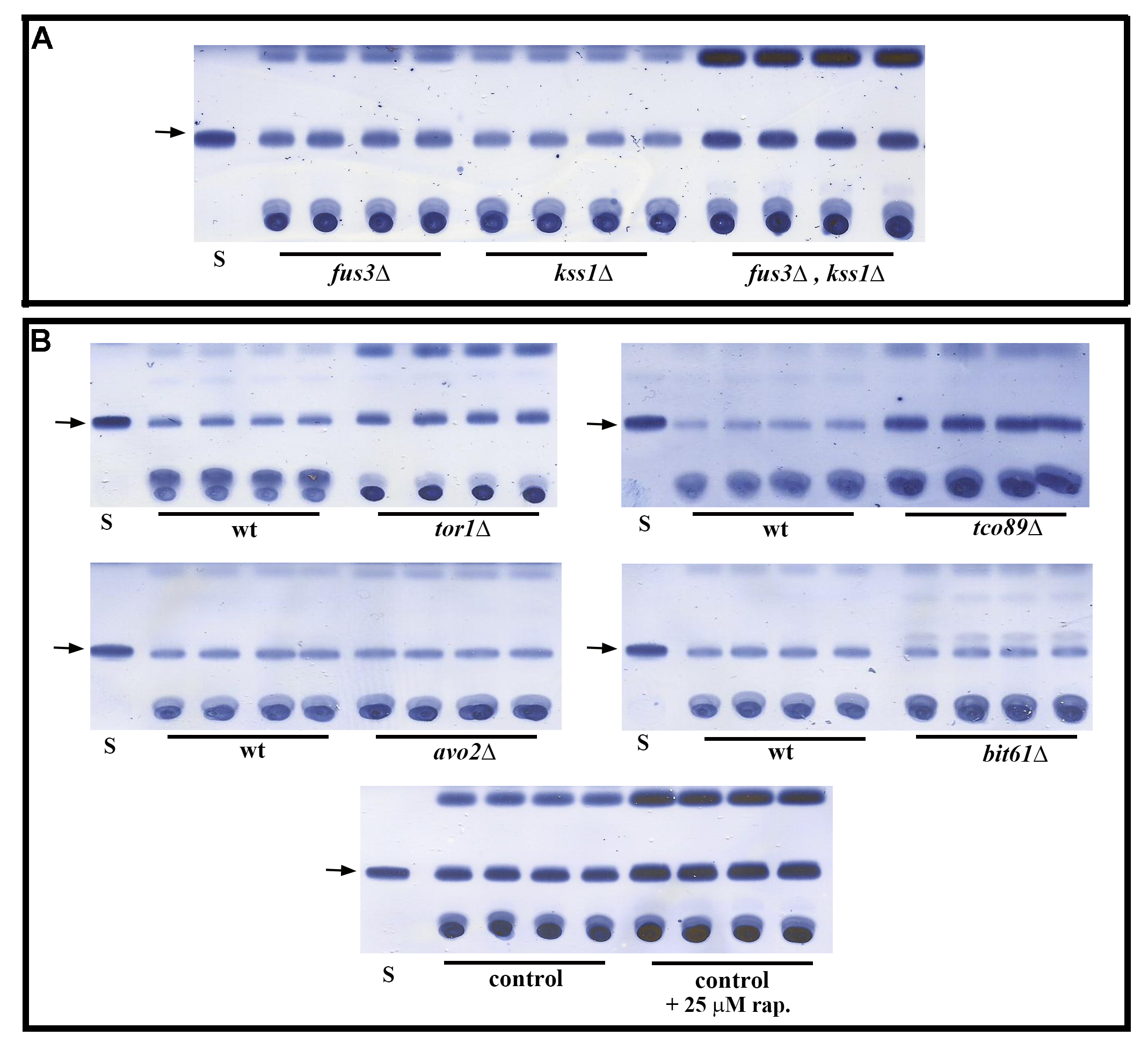

Supplement: S2 Fig — (A) fus3,kss1 double mutants have a higher fat levels than either single mutant. S is 4 μg lard standard, black arrow is triglyceride band, 4 μl volume was used for each sample lane. (B) Mutations in genes encoding TORC1 components (tor1 and tco89) cause an increase in fat storage levels (upper panel), while mutations in genes encoding TORC2 components (avo2 and bit61) do not (middle panel). Treatment of wild type yeast with the TORC1 inhibitor rapamycin causes an increase in fat storage as compared to vehicle only control (lower panel). S is 4 μg lard standard, black arrow is triglyceride band, 4 μl volume was used for each sample lane. (TIF) [file pcbi.1004264.s002.tif]

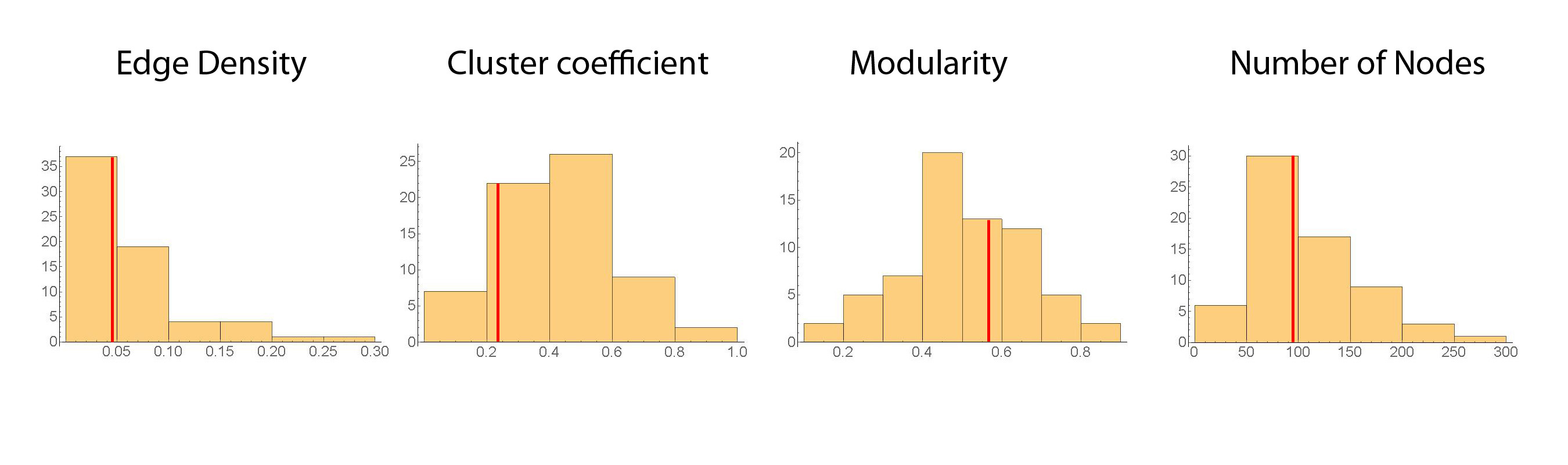

Supplement: S3 Fig — The four histograms show edge density (# edges/# possible edges), cluster coefficient (Cg), modularity (M), and the number of nodes (proteins) for each GO Biological Process category. In each histogram the value of the parameter for the experimental fat storage regulation network is shown by a vertical red line. (JPG) [file pcbi.1004264.s003.jpg]

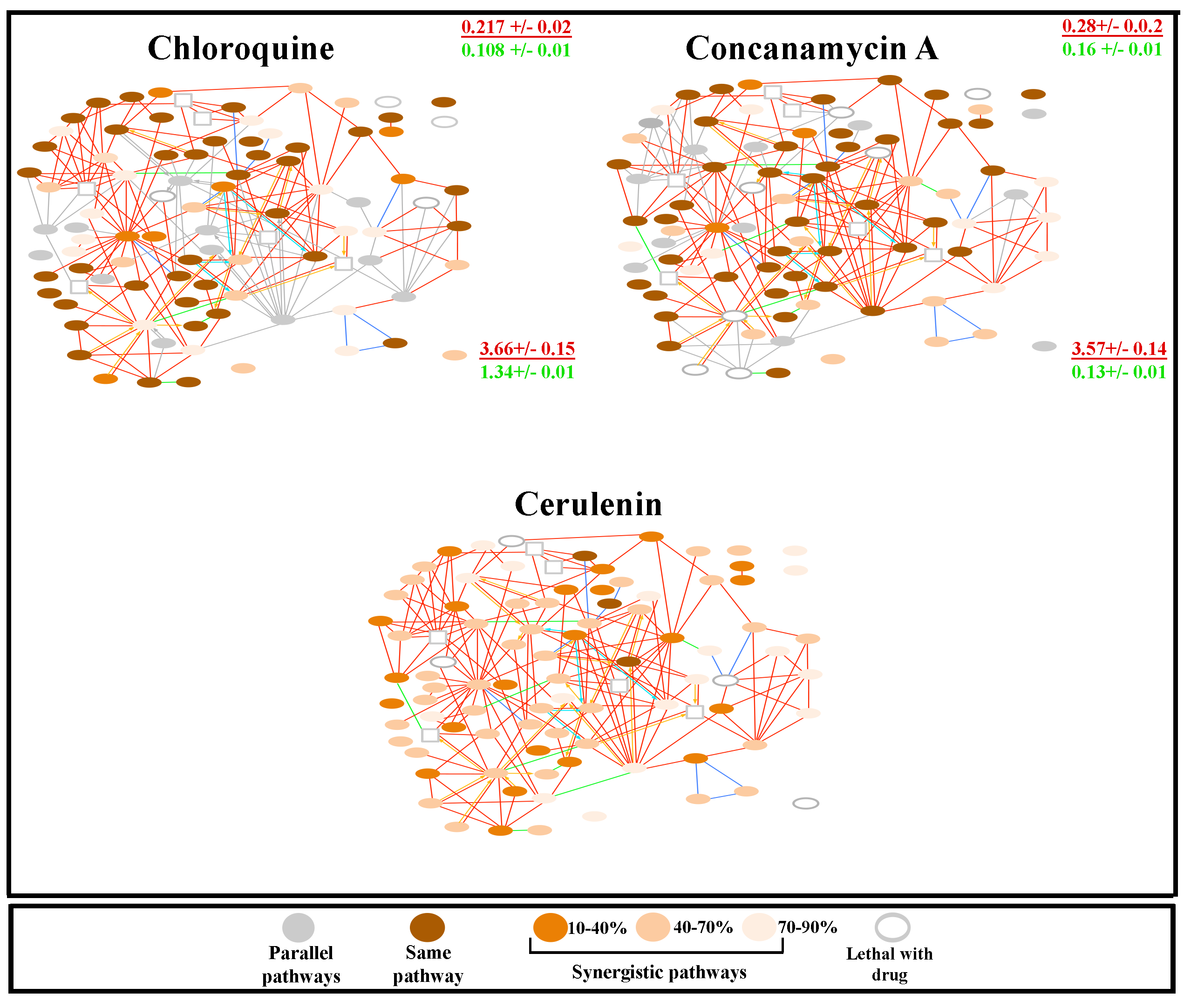

Supplement: S4 Fig — (A) Representations of signaling relationships between drug targets and mutant genes. Networks of interactions between mutants and ChQ, Con. A, and Cer. Protein names are given in larger diagram in Fig 6E. Note that the majority of proteins have signaling interactions with the drugs that range from “same pathway” (no enhancement of drug effect by mutation; dark brown), to different degrees of synergism (enhancement of drug effect by mutation, indicated by different shades of light brown), and there are only a few cases of non-interacting parallel (independent) relationships, in which the drug and mutant effects are additive (light grey). At the right side of panels are indicated the global clustering coefficient (upper) and path length (lower) for a subnetwork of all proteins having a same pathway relationship to that drug (red font), presented over the mean of values from 10,000 generated simulated random networks with the same degree distribution and vertex count (green font). (TIF) [file pcbi.1004264.s004.tif]
