# Supplementary material for: Experimental and Computational Analysis of a Large Protein Network That Controls Fat Storage Reveals the Design Principles of a Signaling Network
Source: PLoS Comput Biol. 2015 May 28;11(5):e1004264. doi: 10.1371/journal.pcbi.1004264 (PMC4447291; doi:10.1371/journal.pcbi.1004264)
Supplement: S1 Text — (DOCX) [file pcbi.1004264.s005.docx]

- **S1 Text**
- **Supplementary Materials and Methods**
- **Starvation study**
- Yeast strains were grown for two days in complete synthetic 2% dextrose media, then transferred to the same media minus glucose. At different time points, fat was extracted and analyzed via TLC and cell viability was evaluated by plating on YPD plates. Four replicas for each time point for a given genotype were obtained, and the rate of fat storage degradation was represented as a trending slope from the average fat band levels for each time point.
- **Growth on different carbon energy sources**
- Yeast strains were grown for two days at 30^°^C in complete synthetic 2% dextrose media. Cultures were then normalized at OD_600_ and three dilutions were made using PBS buffer (0, 1:5, and 1:10). The dilutions were plated using a pinning tool on four types of plates: 3% glycerol, 0.1% palmitate, and 0.1% lard plates.
- **Measuring the rate of *de novo* fatty acid synthesis and glucose consumption**
- Yeast strains were grown for two days in 30^°^C in 3ml complete synthetic 2% dextrose media that was spiked with 10 µCi per ml ^14^C-labeled D-glucose or L-aspartic acid. One ml of culture was spun down, the supernatant removed, and the pellet mixed with 10 ml of scintillation fluid and radioactivity was measured. An additional 1ml sample was used to extract fat from the pellet as described above, and the isolated fat sample was mixed with scintillation fluid and measured for radioactivity. The data was represented as a fat extract signal over total pellet signal in a minimum of three replicas.
- **Drug treatments**
- Yeast strains were grown for 1 day at 30^°^C in 10 ml complete synthetic 2% dextrose media. The culture was spun down, the supernatant removed, and 20 ml fresh media mixed with a given drug was added (10 μM U0126,  μM 25 rapamycin, 0.5% chloroquine, 5 μM concanamycin A, and 50 μM cerulenin). These concentration were chosen due to the fact that they produce the maximum effect on fat levels in wild-type yeast with minimum lethality (data not shown). After a two day incubation at 30^°^C, fat was extracted and analyzed via TLC as described above, and cell viability was evaluated by plating on YPD plates followed by counting the number of emerging colonies as compared to control cultures incubated with only with the DMSO drug vehicle. The data for U0126, rapamycin, chloroquine, and concanamycin A were represented by subtracting the amount of fat added to a given mutant when exposed to a certain drug from the control DMSO treated mutant fat levels. The resulting value was represented as percentage over the amount of fat added to wild-type when exposed to the same drug. For cerulenin, the data were presented as percentage of fat level fraction removed due to drug treatment of mutant over the total fat levels of control DMSO treated mutant. For each treatment a minimum of five replica samples were done. All produced similar fat level reading within 10% standard deviation.
- **Drug specificity**
- The specificity of some dugs was confirmed by the fact that they do not enhance the fat levels of yeast mutants missing their potential targets. An example of this is the inability of U0126 to cause further increase in the fat levels of *mkk1∆* mutants (Fig. 6A), and the inability of rapamycin to cause additional fat increase in mutant missing the Tor complex I obligatory subunit Tco89 (Supp. Table 6). However, we do observe a small increase in fat levels in *tor1∆* mutants incubated with rapamycin, which is likely due to the fact that in yeast Tor complex I can be formed around either Tor1 or Tor2 proteins (S6 Table). Although the ability of both chloroquine and concanamycin A to block vacuolar acidification carried out by the Vma H+-ATPase pump is well known, their respective molecular targets in this large pump complex are not yet fully characterized. However, we did observe that chloroquine failed to produce phenotypic enhancement of *vma5∆* and *vma7∆* mutants, and concanamycin A had no effect on *vma8∆* and *vma13Δ* mutants, thus suggesting that the effect of the two drugs are mediated by different H+-ATPase pumps (S6 Table). We were unable to perform the above stated specificity test on cerulenin due to the fact that mutation in its fatty acid synthase target produce a growth arrest. Nevertheless, the well understood pharmacology of cerulenin and its frequent use in blocking yeast and other cells *de novo* fatty acid synthesis allows for some degree of confidence in its specificity.
- **SUPPLEMENTARY MATHEMATICAL ANALYSIS**

**Mathematical Analysis**

Most of the mathematical analysis was computed using Mathematica (Wolfram Inc.).. Measures of independence between the fat-storage defect and the topological parameters of a given protein were computed using the Blomqvist β, Goodman-Kruskal , Hoeffding D, Kendall tau, and Spearman Rank tests, when applicable. Two variables are considered to be independent when the *p*-value for a test is more than 0.05. Graphics were produced using Mathematica (Wolfram Inc.).

*P(k)* can be formally defined as:

$$P\left( k \right)=\frac{n_{k}}{n}$$

where $n_{k}$ represents the number of proteins in the network of degree *k* and *n i*s the size of the network.

The Power law *P(k)* distribution is approximated by:

$$P\left( k \right)\approx\alpha k^{-\gamma},$$

where $\alpha$ is the y-axis intersection and $\gamma$ is the slope on a log-log plot of *P(k).*

For finite populations, the Poisson *P(k)* distribution can be approximated by:

$$P\left( k \right)\approx\binom{n-1}{k}p^{k}\left( 1-p \right)^{n-1-k},$$

where *n* is the number of proteins, *k* is the number of connections in the network, *p* is the probability that a given node is a member of a node group that has the same number of connection, and $\binom{n-1}{k}$ is the binomial coefficient, calculated by:

$$\binom{n-1}{k}= \frac{\left( n-1 \right)!}{k!\left( n-1-k \right)!},$$

In our estimations, the value of $\alpha$, $\gamma$ and *P* were chosen to minimize a standard sum of squares due to error (SSE).

*C_g_* can be formally defined as:

$C$g$= \frac{3 \times number of triangles}{numbers of triples}= \frac{\Sigma\tau_{\Delta}}{\Sigma\tau}$

where $\Sigma\tau$ is the total number of triplets in the network and $\Sigma\tau_{\Delta}$ is the number of triangles produced by triplets that have three links connecting them.

*M* is formally defined by:

$$M= \sum_{i} (e_{ii}-a_{i}^{2})$$

where *e_ii_* gives the fraction of edges in the network that connect vertices in the same community, and *a_i_* denotes the fraction of edges with at least one end vertex in community *i*.

*L* for a graph that contains a set of nodes *v_i_,* let *d(v_1_,v_2_*) be the shortest distance between nodes *v_1_* and *v_2_*. If two nodes, *v_2_* and *v_1_* cannot be reached from each other, the value of *d(v_1_,v_2_)* is 0. Thus, while *n* is equal to the number of nodes in the graph, the average path length, *L* can be defined as:

$$\frac{1}{n(n-1)}\sum_{i\neq j} d(v_{i},v_{j})$$

The different *C(v)* measurements were obtained using the following formulas:

Betweenness centrality is given by

$$C\left( v \right)= \sum_{v\neq u\neq w\neq v} \frac{\sigma_{wu}(v)}{\sigma_{wu}},$$

where $\sigma_{wu}$ is the total number of paths through w and u and $\sigma_{wu}(v)$ is the total number of paths that also pass through *v* [41].

Closeness centrality is written as

$$C\left( v \right)=\sum_{u \neq v} \frac{1}{d(u,v)} ,$$

*v* is the node of interest that *d* pass through to every other node in the network, and *u* is the central node with lowest total distances to all other nodes [41].

Eigenvector centrality is defined implicitly by

$$C\left( v \right)= \frac{1}{\lambda}\sum_{t} A_{t,v}C(t),$$

where $\lambda$ is the largest eigenvalue of the adjacency matrix, *A*, which is a square matrix whose size is the total number of units used in the matrix that represent our network (in our case 94 by 94 ) [39].

Katz centrality is measured by:

$$C\left( v \right)=\sum_{k=0}^{\infty} \sum_{t} {\alpha^{k}(A)}_{t,v}^{k}$$

where $\alpha$ is called an attenuation factor, which we chose to be half the largest eigenvalue, $\lambda$, of the adjacency matrix, $A$, of our network [40].

- Measures of independence between the fat-storage defect and the topological parameters of a given protein were computed using the Blomqvist ß, Goodman-Kruskal , Hoeffding D, Kendall tau, and Spearman Rank tests, when applicable. Two variables are considered to be independent when the *p*-value for a test is more than 0.05. Graphics were produced using Mathematica (Wolfram Inc.).

**Estimations of the number of low connectivity proteins needed to convert the *P(k)* distribution of the fat storage regulation to a power-law distribution**

To evaluate the strength of the conclusion that our network fits a Watts-Strogatz but not a scale-free model, we asked what changes would be required to convert the network to a scale-free one. The number of proteins with few connections that would be required in order to convert the degree distribution of our network to a power-law was estimated using $\gamma$ values in a range reported by Maslov and Sneppen [67]. The reported distributions represent a broad class of scale-free network whose γ values range from 2.2 to 2.8. In our case, the degree distribution can be fitted by a power law with γ = 2.5 ± 0.3 for a protein connectivity that ranges from 1 to about 100 connections. We also tested against degree distributions of networks generated by the Barabási–Albert scale-free model with a γ that equal 3.0 [68].

For the lower observed range γ a minimum of approximately 110 low connectivity proteins is need for the conversion, while an upper range of γ the number would be 202. However, in the Barabasi-Albert model, the number of required low connectivity proteins would be close to 266. These estimates are based on standard sums of squares due to error that is within the range of our network Poisson distribution. Furthermore, to increase the stringency of our estimates, the low connectivity proteins were not chosen from a distribution that would favor our conclusion, but rather were chosen in an optimal manner that fit the required power-law distribution more favorably.
